# The Complexity of Remote Learning: A Neuroergonomical Discussion

**DOI:** 10.3389/fnbot.2022.842151

**Published:** 2022-04-13

**Authors:** Federico Cassioli, Michela Balconi

**Affiliations:** ^1^International Research Center for Cognitive Applied Neuroscience (IrcCAN), Università Cattolica del Sacro Cuore, Milan, Italy; ^2^Research Unit in Affective and Social Neuroscience, Department of Psychology, Università Cattolica del Sacro Cuore, Milan, Italy

**Keywords:** cognitive neuroscience, neuroergonomics, remote working, environment, psychophysiology

## The Inherent Complexity of Learning

In this work, we aimed at affirming the inherent complexity of learning processes and the consequent benefits derived from a *multi-layer cascade* approach that considers heterogeneous disciplines and furnishing actionable best practices, for the designing of a learning experience in an organization.

Since disciplines, at different scales, bring together heterogeneous knowledge, we advocate for an integration of them. The various types of learning (i.e., non-associative, associative, perceptual, and motor) can be explored to understand the development, storage, and recall of memories, using molecular, cellular, and systems data. Neurobiologically, learning corresponds to functional and structural changes in the synapses at a variety of loci, throughout the central nervous system (e.g., Kopec et al., 2007). These modifications consist in post-translational variations of proteins in the synaptic site, connected to synaptic plasticity, *via* interrelated changes in biochemistry, physiology, and subcellular redistribution. Evidence from laboratory animals strongly supports this relation (Lynch et al., 2007), even though a direct causality linked to behaviors is an open discussion (Mao et al., 2011).

At a higher scale, modern brain imaging procedures have provided information about the activation of brain regions such as the *limbic system*, the *cerebellum, striatum, amygdala*, and other motor or sensory systems, which encode and store information into long-term memory (Markowtich, 2005). These brain areas contribute to the development of competence and skills in a worker.

Parallelly, assuming a synthetic rather than reductionist perspective, learning does not necessarily consist of specific responses made to certain stimuli, conversely learning and memories should not be considered stable and definite.

Finally, a distinction between learning and performance is needed. Even if highly related, these two concepts do not perfectly match. For example, latent learning could be obscured by a performance factor (e.g., a motivation deficiency can inhibit goal-oriented behaviors), such as attention, sensory-receptor sensitivity, motivation, and arousal.

Recent human-based studies on cognition regarding training and memory added value to the research line of learning. In this light, human brain functions are thought within an environment, together with a *dynamic relation*, which is at least partially socially constructed, with work and technology. The application of techniques such as fMRI, fNIRS, electroencephalography (Balconi and Molteni, 2016; Belkhiria and Peysakhovich, 2020; Nozawa et al., 2021), together with behavior-oriented approaches, allowed the development of *neuroergonomics*. Its main advance consists of the assumed bottom-up, situational-oriented perspective. *Via* these techniques, combined with novel computational modeling (Cassioli and Balconi, 2020), strategies for effective training can be assessed (e.g., Kenny and Power, 2021), by comparing the related underlying neural processes.

For example, the impact of digital technology on learning processes in the organizational framework is a crucial key point, even if it is still mostly unexplored. Novel organizational tools may determine different behaviors and novel responses, with significant consequences on the training efficacy.

Since the learning process is heavily influenced by the employed medium and environmental factors, we propose neuroergonomics as that perspective that uses neurobiological evidence, by considering stressors and well-being, and focuses on the cognitive and affective dimensions.

**Figure 1:**
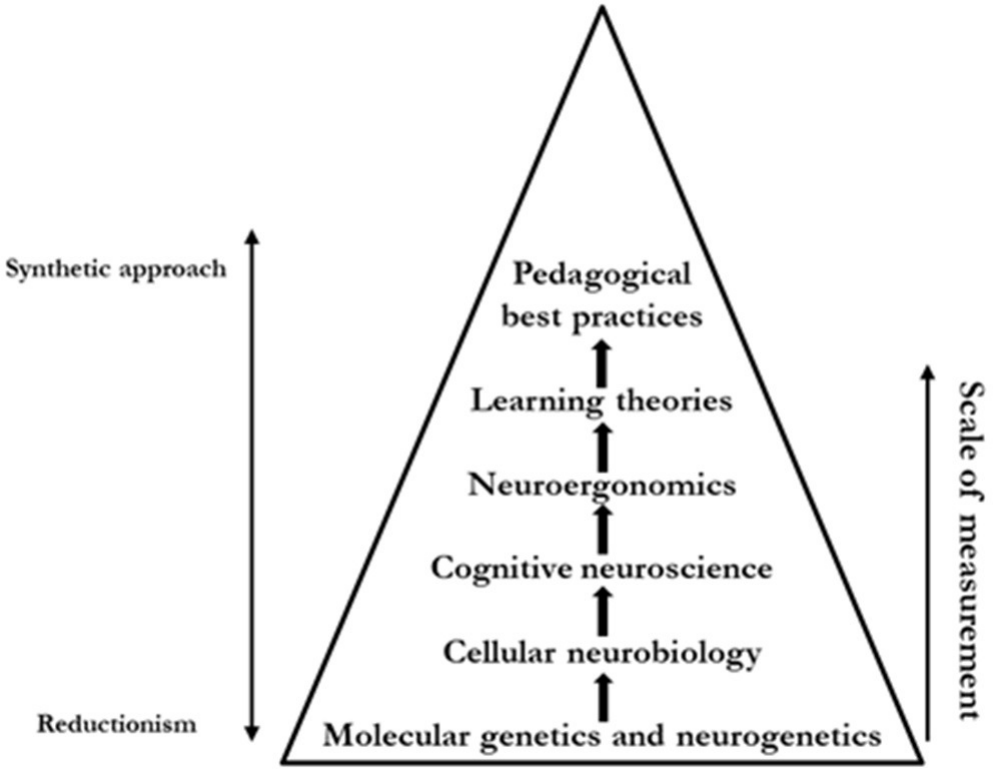
The representation of a “*multi-layer cascade*” approach concurring in the development of applicable best practice regarding the learning process.

Each of these presented paradigms composes a layer that concurs in the development of applicable knowledge for the organization, enriching learning theories. We firmly believe that the consideration of multiple sources of information could help the development of best practices. A scheme is reported in Figure 1. A multi-layer cascade orientation refers to that epistemological approach we advocate for that sees cross-contamination between different disciplines as valuable. Every layer is enhanced by insights derived from previous layers and leads in the direction of the development of actionable best practices.

Despite this promising opportunity, scarce evidence, which, for example, considers neurocognitive and emotional parameters, was gathered, with limited applications in the pedagogy of education for heterogeneous tasks or settings.

## Neuroergonomics in The Organization

### An Operational Definition of Neuroergonomics

Neuroergonomics investigates the neural bases of mental and physical functions, in applied settings, such as work, transportation, and health care (Parasuraman et al., 2012). It is defined as the study of human brain function in relation to work, together with technology (Parasuraman, 2003). Real-world contexts are assessed within a framework where human intentions, actions and behaviors are considered interdependent with the environment (Dehais et al., 2020).

The emergence of new digital tools, together with the ubiquity of technology, is known to play a transformative role within organizational processes. For this reason, neuroergonomics, by considering the neurocognitive and the physical dimensions, can support the investigation of the complex relationship between workers and learning mediated by technology.

From an epistemological and methodological perspective, neuroergonomics in the organization assumes that the effects of computer-mediated interaction are mirrored at psychological, neurocognitive, and physiological levels. Biomarkers, which refers to psychophysiological states, are then selected to assess the cognitive dimension, by considering attentional processes, executive functions and mental workload, and affective states, such as arousal activity, emotional categorization, cardiovascular fitness, and resiliency (Balconi et al., 2019; Crivelli et al., 2019; Getzmann et al., 2021). Neuroergonomics, merging neurobiological and neurocognitive evidence, with quantitative-based behavioral analysis, can be employed to record outcomes, and provide feedback on learning. In this sense, neuroergonomics represents an approach that supports a deeper understanding of workers and their behaviors and facilitates the reaching of their maximum potential. To design a training in an organization, the added value of neuroergonomics might be substantial. For this reason, aspects such as environmental factors and employed technologies (i.e., *learning management system*) should be considered.

### Environmental Factors and Learning Experience

As mentioned above, training should be considered from a holistic perspective, as people act within a learning environment. In fact, as Kaplan and Kaplan (2003) argued, environment has a profound effect on human cognition, behavior, and well-being. Within the framework of *attention restoration theory* (Kaplan, 1995), environmental processes play a significant role in the mental fatigue levels and in how restorative settings can foster recovery. Many factors can intervene in the learning experience, such as lighting. In fact, a natural source seems to influence the limbic system, with positive impacts on mood, sleep, and cognitive performance (Samani et al., 2013). Other parameters that might interfere are room temperature, environmental noise and many more. For example, the environmental *restorativeness* should be as well considered because it elicits emotional, cognitive, and physiological responses. Furthermore, spatial arrangement helps defining the individual *place identity*, which is a good driver for performance and fosters the sense of community (Knight and Haslam, 2010) within the organization.

### Technology Disruption and Learning

Technology-mediated interactions face a further challenge. The technology disruption we experienced, calls for a sophisticated analysis between different modalities (i.e., face-to-face vs. remote) which impact the learning process. Since organizations extremely often make decisions based on available resources, when administering a training course, only the most efficient solution should be considered (Waytz and Gray, 2018). Actual evidence appears troublesome. Overall, there is a small understanding of how virtual contexts work on psychological dimensions and how they impact work performance.

Online communication is sometimes linked to lower empathy (Wellman et al., 2006). Also, remote training, often explored on students or healthcare workers and is reported to present both pros and cons. In fact, remote training allows no spatial constraints, flexibility, and the possibility to easily access the available resources (Hoyer, 2006). It could be then inferred that *lifelong learning* in workers could be facilitated by distance learning. Unfortunately, past research has shown that the proliferation of open courseware (e.g., MOOCs) tends to exacerbate individual differences, which are explained by training motivation (Horrigan, 2016). Furthermore, the affordances of technologies and their effects are not neutral (Houlden and Veletsianos, 2019) and should also be contemplated. In addition, since remote settings are not always designed for learning scopes, they might present features which are not optimal for training. Conversely, face-to-face interactions might result challenging and stressful because of personal factors (i.e., anxiety trait), with significant performance penalties.

Further evidence should be gathered considering setting (e.g., face-to-face vs. remote) conditions and their effects on the learning outcome in workers. Authors think that both cognitive neuroscience and neuroergonomics contributions are expected to deliver more evidence in the coming era.

Moreover, research before SARS-CoV-2 highlighted a mild positive relationship between employees' engagement levels and time spent in remote conditions. Data showed that, when spending up-to-20% of the time from distance, employees tend to be more motivated and attached to the company (Gallup Organization, 2013). Moreover, the outspread of covid-19 limited physical proximity and imposed stay-home restriction and remote- and/or smart-working, accelerating the digital transformation. Data indicated that people worked fewer hours or even temporarily stopped working at their job (e.g., Gallup Organization, 2021), showing decreased engagement levels in the daily activities and experienced (>40%) daily worries and stress. In this light, being unengaged employees means not having psychological attachments, with a lack of energy and passion and a tendency to be resentful that your own needs are not being met and thus avoiding the acquisition of skills that strengthen the performance and boost a company's success.

Finally, we believe that remote learning on online platforms is often presented to trainees *via* reward-oriented platforms. Risks of an attentional shift from the course content to the activity completion time (often offered in percentage) are more than plausible. Indeed, workers might be wrongly rewarded not by the skills or knowledge they acquire but by other factors, such as the pace they keep. As previous scientific evidence suggests, an inherent reward tends to be a stronger psychological driver for a certain behavior compared to an external one.

In the following paragraph, we briefly present some recommendations which could be considered by practitioners when designing a learning module within an organization.

## Conclusion: Recommendations and Future Directions

As expounded, neuroergonomics can be operationalized as the study of human brain function in relation to work and technology.

To manage the inherent complexity of learning, we now propose to consider the following recommendations which should be considered when designing a learning experience in an organization.

- *Applying a multi-layer cascade approach*. Based on current scientific evidence-based knowledge from neurobiology, cognitive neuroscience and neuroergonomics, best practices should be developed and shared with trainers and learners. The underlying neurobiological principles shape the pedagogy of learning.
- *Test, implement, test*. Both reductionist and synthetic approaches have shown to provide useful insights. Therefore, we should design research that investigates how digital tools impact human wellbeing, work performance, output quality and learning. Since existing evidence is troublesome, a better comprehension of its effects on the physiological, cognitive, and affective dimensions is needed. According to the authors, small evidence has been gathered on real-world contexts so far. Neuroergonomics represents a good perspective where the evaluation of learning processes is considered interdependent with human behaviors, intentions, and the environment.
- *Simplicity is seductive but often wrong*. Learning abilities differ due to age, role, motivation, mental state, and environmental factors. Learning happens in all ages of a living organism, although not always under equal conditions. Remote and face-to-face settings both present pros and cons. Siding with a certain one, until sufficient evidence is gathered (see *ii*.), denies the inherent complexity of learning.
- *Assuming an equality between behavior and learning is wrong*. Streaming and completing a training course does not necessarily convert into the acquisition of competence. When computing the efficiency of a technology system, not pondering human factors might undermine the ultimate purpose.
- *Learning management systems should be competence-oriented*. Online platforms for learning courses (e.g., MOOC) should not be reward-based considering completion, but knowledge- and competence-oriented. Trainees should develop a focus on the abilities they are learning, understanding how those skills might be pragmatically valuable for them.
- *Knowledge and competence are also socially constructed*. Remote vs. face-to-face training activities should be considered based on trainees, course content, situational and environmental factors. Blended solutions could represent a possibility.
- *Acknowledge the existence of miscellaneous unaccountable phenomena*. Culture, digital divide, data security, and privacy are just a few of the many issues which should be further considered when designing a training course.

In this work, we highlighted how a multi-layer cascade approach represents an attempt for an overall comprehension of learning processes. Despite all, this study presents limitations. We did not consider other factors such as the learning content, individual traits, and personal predisposition. Future studies could investigate the impact of these dimensions as well.

To conclude, future lines of research should focus on the impact of technology disruption on human beings at work and consider side factors by integrating contributions from heterogeneous domains. Ultimately, beyond the chosen medium, trainers, supported by scientists, should enable learners to obtain gratification from the doing, not the results.

## Author Contributions

FC and MB theoretically designed the framework and together finalized the actual version of the article. All authors contributed to the article and approved the submitted version.

## Funding

This work was supported by D.3.2. Fund (ricerche di particolare interesse per l'Ateneo) 2020: Behavioural change: prospettive per la stabilizzazione di comportamenti virtuosi verso la sostenibilitá at Universitá Cattolica del Sacro Cuore.

## Conflict of Interest

The authors declare that the research was conducted in the absence of any commercial or financial relationships that could be construed as a potential conflict of interest.

## Publisher's Note

All claims expressed in this article are solely those of the authors and do not necessarily represent those of their affiliated organizations, or those of the publisher, the editors and the reviewers. Any product that may be evaluated in this article, or claim that may be made by its manufacturer, is not guaranteed or endorsed by the publisher.
